# Impaired aspirin-mediated platelet function inhibition in resuscitated patients with acute myocardial infarction treated with therapeutic hypothermia: a prospective, observational, non-randomized single-centre study

**DOI:** 10.1186/s13613-018-0366-x

**Published:** 2018-02-21

**Authors:** Florian Prüller, Oliver Leopold Milke, Lukasz Bis, Friedrich Fruhwald, Daniel Scherr, Philipp Eller, Sascha Pätzold, Siegfried Altmanninger-Sock, Peter Rainer, Jolanta Siller-Matula, Dirk von Lewinski

**Affiliations:** 1 Clinical Institute of Medical and Chemical Laboratory Diagnostics, University Hospital Graz, Graz, Austria; 2 Medical University of Graz, Graz, Austria; 3 Department of Cardiology, Intensive Care Unit, University Hospital Graz, Auenbruggerplatz 15, 8036 Graz, Austria; 4 Department of Internal Medicine, Intensive Care Unit, University Hospital Graz, Graz, Austria; 5 Department of Cardiology, Vienna General Hospital, Wien, Austria

**Keywords:** Aspirin, P2Y12, Resuscitation, Platelet function

## Abstract

The majority of resuscitated patients present with underlying cardiac disease, and out of these myocardial infarction is most common. Immediate interventional treatment is recommended and routinely requires dual antiplatelet therapy including aspirin and a P2Y12-inhibitor. Therapeutic hypothermia or target temperature management is also recommended in these patients. Cardiogenic shock as well as reduced body temperature impacts platelet reactivity and its medical inhibition. The study aims to quantify aspirin- and P2Y12-mediated platelet inhibition in patients presenting with myocardial infarction and cardiopulmonary resuscitation. Twenty-five resuscitated patients were enrolled in this prospective, observational, non-randomized single-centre study. These patients were compared to 77 matched controls from the ATLANTIS-ACS database of non-resuscitated patients with myocardial infarction. Platelet function testing was performed by light transmittance aggregometry. Aspirin reactivity was monitored by inducing platelet aggregation with collagen and arachidonic acid, respectively. P2Y12 inhibition was recorded by stimulation of platelet aggregation with adenosine diphosphate. To quantify the overall platelet response, thrombin receptor-activated peptide was used. Aspirin-mediated platelet reactivity decreased significantly in resuscitated patients during the first days and was significantly weaker on day 3 (collagen AUC 253.8 (122.7–352.2) vs. 109.0 (73.0–182.0); *p* = 0.022). P2Y12-mediated platelet inhibition was also impaired in resuscitated patients on day 3 (mean ADP AUC (IQR): CPR 172.1 (46.7−346.5) vs. control 43.9 (18.9–115.2); *p* < 0.05). Aspirin- and P2Y12-mediated platelet inhibition is impaired in resuscitated patients treated with therapeutic hypothermia. On day 3, we recorded lowest inhibitory effects of both drug types and patients might be at particular risk at that time. Potentially, intravenous aspirin and P2Y12 inhibitors might still supply a more predictable and stable platelet inhibition.

**Electronic supplementary material:**

The online version of this article (10.1186/s13613-018-0366-x) contains supplementary material, which is available to authorized users.

## Background

Therapeutic hypothermia as targeted temperature management of 32–36 °C for 24 h is still recommended for patients after cardiopulmonary resuscitation (CPR) to improve neurological outcome [1]. Approximately 60% of all out-of-hospital cardiac arrest patients have suffered acute myocardial infarction (AMI), and the majority of these patients undergo primary percutaneous intervention (PCI) and therefore require dual antithrombotic therapy.

Dual inhibition of platelet function with aspirin and a P2Y12 inhibitor is recommended as soon as possible in STEMI patients. Timing in NSTEMI patients is controversial, but therapy should be initiated no later than in the cathlab after confirmed diagnosis of NSTEMI [2]. Early administration of platelet inhibitors is necessary as highest risk of stent thrombosis (ST) is reported within the early phase after stent implantation.

There is evidence for an increased risk of stent thrombosis after PCI despite administration of antiplatelet drugs in resuscitated patients treated with therapeutic hypothermia [3, 4]. Underlying mechanisms of bioavailability are still unclear; however, both reduced gastrointestinal absorption due to cardiogenic shock and delayed metabolism as well as an accelerated platelet turnover due to inflammation might cause altered platelet inhibition and plasmatic coagulation.

Hypothermia itself is decreasing coagulation and platelet aggregation. Plasmatic coagulation is blunted at reduced body core temperatures as clotting factor activity is reduced to ~ 50% [5] and both clotting time and clot formation time are prolonged in patients undergoing hypothermia to 36, 34 and 32 °C due to neurosurgery [6]. In addition, thrombocyte count is also decreased after induction of hypothermia.

Most data on platelet function in hypothermic patients are available for patients receiving clopidogrel although an increasing number of studies reported data of patients treated with new P2Y12 inhibitors (ticagrelor and prasugrel), which are recommended in recent ACS guidelines [2, 7, 8]. None of these studies described aspirin-mediated effects on platelet reactivity and only one recent small trial focussed on these effects, yet [9]. We therefore aimed to test platelet function in a prospective trial for all resuscitated ACS patients transferred to our centre, evaluating both aspirin- and P2Y12-mediated platelet function inhibition. We compared the efficacy of the medication within the study group and to a matched control group of patients with ACS without resuscitation and therapeutic hypothermia.

## Methods

**Fig. 1:**
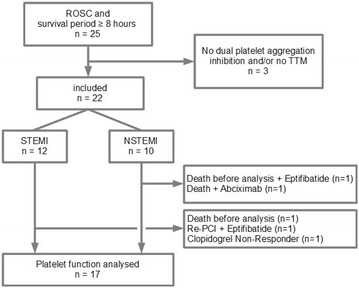
Flow diagram of inclusions and exclusions for patients with return of spontaneous circulation

A total of 25 out of hospital resuscitated patients with the diagnosis of troponin-positive ACS interventionally proven coronary heart disease (NSTEMI or STEMI) and survival until the morning after the index event were consecutively included in this prospective, observational, non-randomized single-centre study (Fig. 1). Three patients were excluded as no therapeutic hypothermia was applied based on a short period until ROSC and anticipated early extubation. One of these patients did not receive dual platelet inhibition. Five of the remaining 22 patients had to be excluded according to the protocol. Two patients died before analysis of platelet inhibition, 3 patients received GpIIb/IIIa treatment predefined as exclusion criterion because P2Y12-specific analysis of platelet inhibition is not feasible with this treatment. And one further patient was clopidogrel non-responder and was not included, too, since this patient had to be switched to a new P2Y12 inhibitor within the study period.

Patients were enrolled from May 2015 to July 2016. We defined the following three inclusion criteria: (1) age > 18 years, (2) resuscitation due to acute myocardial infarction, (3) intrahospital survival for at least 8 h. Clinically, significant bleeding leading to discontinuation of antiplatelet therapy was used as an exclusion criterion. These patients were compared to 77 matched controls from the ATLANTIS-ACS database of non-resuscitated patients with myocardial infarction. Since platelet function was only measured once in each patient of the ATLANTIS-ACS cohort, control groups for the three first days were assembled of different but matched control patients. The study complied with the Declaration of Helsinki, and the protocol was approved by the Local Ethics Committee of the Medical University of Graz (No. 27-290 ex 14/15) and registered at clinicaltrials.gov with the ID NCT02914795.

All patients received intravenous aspirin (150–300 mg) before catheterization and loading doses of either clopidogrel (600 mg), prasugrel (60 mg) or ticagrelor (180 mg) via gastric line right after percutaneous intervention. However, 4 patients were on previous treatment with clopidogrel (*n* = 3) or ticagrelor (*n* = 1). All other patients enrolled were P2Y12 naïve at the time of ACS. Patients who underwent cardiopulmonary resuscitation (CPR) were treated according to the local standard operating procedure protocol including intravascular cooling (Thermogard, Zoll) initiated immediately after patients’ arrival in the cath lab. Cooling objective was a reduction in body core temperature to 33 °C. Patients were maintained on this temperature for a total of 24 h with a rewarming period of 20 h with a heating rate of 0.2 °C/h. Endotracheal intubation, mechanical ventilation, deep analgosedation and relaxation were applied in all patients.

An age- and gender-matched control group of 77 hemodynamically stable patients was extracted from the ATLANTIS-ACS trial database. These patients had myocardial infarction without resuscitation. Patients’ baseline characteristics are listed in Table 1. The matching ratio was 1:3.5 for the control population.

**Table 1:** Characteristics at admission

*n* (%) or median (IQR)	Study group (*n* = 22)	Control group (*n* = 77)	*p*
Characteristics			
Age (years)	65.0 (50.0–75.8)	64.3 (59.3–72.7)	0.983
Male sex	19 (86.4)	59 (76.6)	0.324
STEMI	12 (54.5)	52 (67.5)	0.261
Coronary angiography			
1—vessel disease	10 (45.5)	17 (22.1)	┐
2—vessel disease	7 (31.8)	31 (40.3)	0.088
3—vessel disease	5 (22.7)	29 (37.7)	┘
Intervention			
Stent implantation	17 (77.3)	74 (96.1)	0.013
Laboratory parameters			
Platelet count (G/l)	188.0 (164.5–223.0)	209.0 (172.0–244.0)	0.630
pTT (s)	47.1 (33.4–107.0)	31 (40.3)	0.009
INR (1)	1.2 (1.2–1.6)	29 (37.7)	0.000
Risk profile/medical history			
Body mass index (kg/m^2^)	28.9 (26.1–30.9)	27.2 (25.2–30.9)	0.755
Alcoholic disease	2 (9.1)	19 (24.7)	0.511
Nicotine abuse	10 (45.5)	38 (49.4)	0.154
Arterial hypertension	11 (50.0)	62 (80.5)	1.000
Diabetes mellitus	3 (13.6)	15 (19.5)	0.481
Hyperlipidemia	9 (40.9)	37 (48.1)	0.385
Peripheral vascular disease	4 (18.2)	3 (3.9)	0.010
Cerebral ischemia	4 (18.2)	0 (0.0)	0.000
Myocardial infarction	4 (18.2)	10 (13.0)	0.273
Coronary angiography	8 (36.4)	11 (14.3)	0.003

*IQR* interquartile range and *STEMI* ST elevation myocardial infarction

Blood samples for platelet function testing were taken every following working day morning for 7 days in the resuscitation group and only once per patient in the control ACS group within the first 3 days after the index event.

Platelet function testing was performed by light transmittance aggregometry (LTA) on a Chronolog 700 Aggregometer (Chronolog Corp., Havertown, PA). Aspirin reactivity was monitored by inducing platelet aggregation with 2 μg/ml collagen and 0.5 mmol L^−1^ arachidonic acid (AA, Chronolog Corp., Havertown, PA), respectively. P2Y12 inhibition was recorded by stimulation of platelet aggregation with 10 μmol L^−1^ adenosine diphosphate (ADP) (Sigma-Aldrich, Vienna, Austria). To quantify the overall platelet response, 40 μmol L^−1^ thrombin receptor-activated peptide (TRAP) (Bachem, Weil/Rhein, Germany) was added. Results were displayed using the Aggrolink 8.1.2.2 software package (Chronolog Corp., Havertown, PA)

Data are given as median (interquartile range). Statistical analysing was performed using the Kruskal–Wallis and the Mann–Whitney *U* tests. *P* values below 0.05 were considered as statistically significant. Power calculation was based on estimated ADP AUC values of 120 and 80 in control and study groups, respectively, with a standard deviation of 40, an alpha of 0.05 and a power of 0.8. 10% drop out was calculated.

## Results

Demographic data were quite similar in both groups (Table 1) and within the group of resuscitated patients divided by the P2Y12 inhibitor used (Additional file 1: Table s1), although patients with resuscitation had less 3-vessel disease and PCI at index event was performed in fewer patients.

**Fig. 2:**
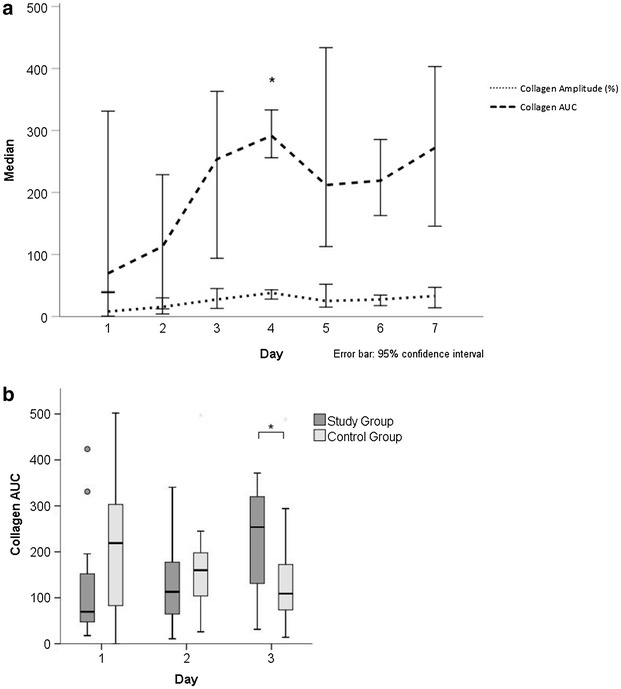
Median collagen AUC and amplitude for the first 7 days after admission in the study group (**a**) and comparison of AUC in study group and control group during the first 3 days (**b**)

Aspirin-mediated platelet reactivity inhibition (judged on arachidonic acid and collagen response, respectively) decreased significantly over time during the first days. There was strong and sufficient platelet inhibition on day 1 with median collagen values of 8.0 (6.0–25.0) and median AUC values of 69.5 (46.7–195.6). This inhibition declined to 33.0 (17.0–47.0) or AUC of 272.0 (148.0–389.9) on day 4 indicating significantly less platelet inhibition with aspirin (Fig. 2a). Arachidonic acid showed a robust inhibition in both groups (data not shown) with a trend towards decreasing from day 1 (32; 13–55) to day 7 (32; 28–44) in the CPR group. There was no significant correlation between the preclinical dose (*r* = 0.323; *p* = 0.282) or the time between initial dose of intravenous aspirin and first analysis the next morning (*r* = 0.009; *p* = 0.96). Compared to control patients, aspirin-mediated platelet inhibition decreased in resuscitated patients during the first 3 days [collagen AUC; day 1: 69.5 (46.7–195.6), day 2: 113.0 (64.3–199.5), day 3: 253.8 (122.7–352.2)], whereas collagen AUC decreased in the control group indicating stronger aspirin-mediated inhibition [219.0 (80.5–334.5), 160.0 (102.0–202.0), 109.0 (73.0–182.0)] for days 1–3, respectively (Fig. 2b). On day 3, there was a significantly reduced platelet inhibition in the CPR group (collagen AUC: *p* = 0.022; collagen amplitude: *p* = 0.017) compared to control.

**Fig. 3:**
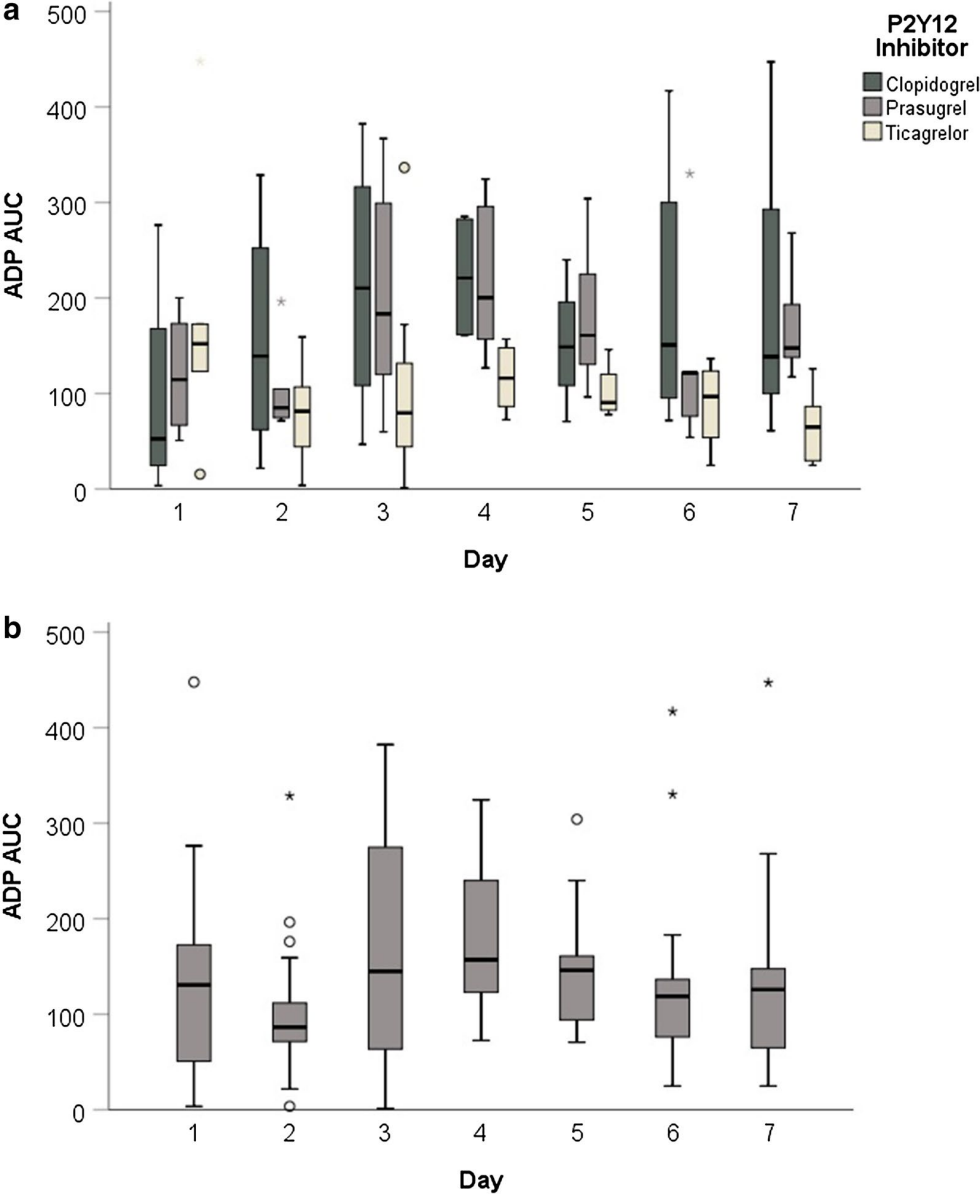
Median ADP AUC for the first 7 days after admission. **a** Itemized by P2Y12 inhibitors. **b** For the entire study group

**Fig. 4:**
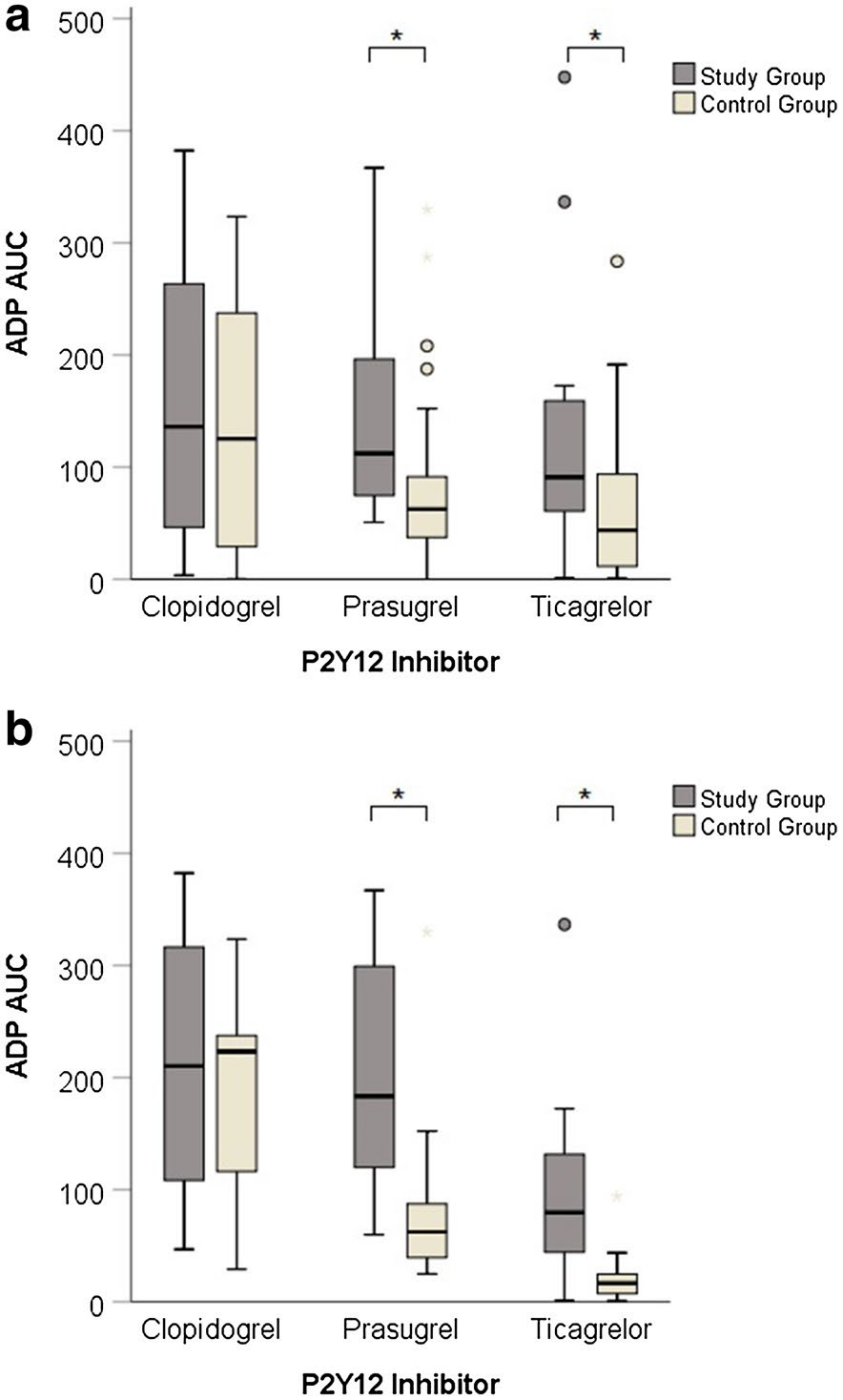
Median ADP AUC by P2Y12 inhibitors. Pololed analysis of first three days (**a**) and day 3 alone (**b**)

With respect to P2Y12 inhibitors, we observed reduced platelet inhibition (judged on ADP response) in the pooled analysis of the first 3 days as well as on day 3 alone in the CPR group (pooled analysis for the first 3 days: Mean ADP AUC (IQR): CPR 102.0 (75.4–179.5) vs. control 59.7 (19.0–124.8), *p* < 0.05, see Fig. 3a; day 3: Mean ADP AUC (IQR): CPR 172.1 (46.7–346.5) vs. control 43.9 (18.9–115.2); *p* < 0.05, see Fig. 3b). However, the degree in platelet reactivity inhibition within the group of resuscitated patients was not changed over time and did not show significant differences in the subgroups for clopidogrel, ticagrelor and prasugrel (Fig. 4).

**Fig. 5:**
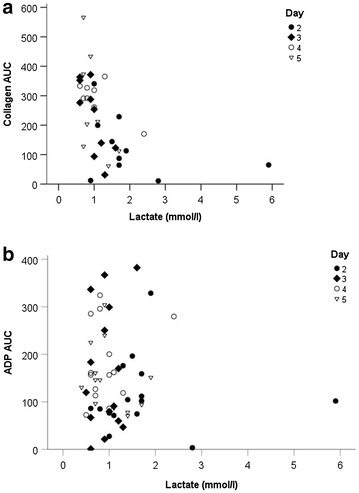
Correlation of serum lactate levels with **a** collagen AUC. **b** ADP AUC

Cardiogenic shock with gastroparesis might play a significant role in delayed absorption of aspirin or P2Y12 inhibitors applied via gastric line. We therefore performed Spearman correlation analysis (r) with respect to markers of cardiogenic shock or predictors of poor outcome after resuscitation. Serum lactate, however, showed a pronounced negative correlation with collagen AUC (Fig. 5a, − 0.654 and *p* < 0.001) but no significant correlation with ADP AUC (Fig. 5b, *r* = − 0.082; *p* = 0.542). SOFA score, too, did show a negative correlated with collagen AUC (*r* = − 0.441; *p* = 0.01) but not for ADP AUC (Additional file 1: Fig. S1, *r* = 0.020; *p* = 0.890, respectively; n.s.).

## Discussion

The data presented indicate that (1) aspirin-mediated inhibition declines significantly within the first days after resuscitation. (2) Aspirin-mediated inhibition is reduced in CPR patients compared to control on day 3. (3) P2Y12-mediated platelet inhibition by ticagrelor and prasugrel is reduced in resuscitated patients. (4) The effect is transient and also most pronounced on day 3.

Previous research focussed on P2Y12-mediated platelet inhibition in resuscitated patients with therapeutic hypothermia and most studies revealed reduced or blunted effects of these drugs in this setting, indicating a potentially increased risk of acute stent thrombosis. Aspirin-mediated platelet inhibition, however, was neglected despite the fact that aspirin is the ubiquitous cornerstone of dual platelet inhibition in ACS. We therefore focussed on analysing aspirin-mediated platelet function inhibition in this prospectively recruited cohort.

Data on the prevalence of high on-aspirin platelet reactivity (HPR) are highly heterogeneous ranging from 0 to 57% [10]. This is due to the variety of laboratory methods and cut-off values used, cohorts of different diseases analysed and the time point of analysis within the trials. We used light transmission aggregometry (LTA) the gold standard method to monitor platelet function inhibition [11].

Aspirin response is judged on collagen and arachidonic acid stimulation and P2Y receptor inhibition on ADP stimulation, respectively. Other frequently used platelet function tests such as PFA-100 are influenced by platelet count and circulating von Willenbrandt factor (vWF), and hypothermia itself reduces platelet count and increases vWF expression. PFA-100 values rather reflect vWF activity than platelet function [12] and were therefore not used for our analysis.

HPR was target in many studies ranging from healthy volunteers [13] to disease specific cohorts with cardiovascular [14] or hematologic [15] diseases and to large all-comers “real world” cohorts [16]. However, there is only one recent study describing aspirin-mediated platelet inhibition in resuscitated patients and therapeutic hypothermia [9]. This trial prospectively tested for differences between oral or intravenous administration of aspirin in CPR survivors treated with therapeutic hypothermia of 33 °C for 24 h using surface cooling. Intravenous aspirin was more likely to result in therapeutic platelet inhibition on day three compared to oral aspirin.

Four reasons might underlie the blunted inhibitory effect of aspirin. First, crushing tablets before administration via the gastric line might impair their effects. Second, gastric paresis and reduced gastric and intestinal perfusion due to reduced cardiac output and centralized circulation might reduce or delay effects. Third, hypothermia itself impacts platelet inhibition. Fourth, resuscitation with consecutive inflammation might directly affect platelet reactivity.

Although usually administered as coated tablets, the used aspirin formula (Thrombo-ASS; G.L. Pharma, Lannach, Austria) is supposed to be chewed in myocardial infarction anyway and aspirin is known to maintain platelet function inhibiting effects after being crushed. However, gastroparesis is a ubiquitous finding in resuscitated patients within the first hours and days after resuscitation and neuroprotective hypothermia in resuscitated patients also requires relaxation und most of the patients have poor hemodynamics even after successful percutaneous intervention. These general features might blunt reabsorption of any orally administrated drug, reduced absorption of orally administered drugs is common in sedated patients, and this effect is further aggravated by therapeutic hypothermia [17].

Only emerging evidence is available that resuscitation itself or corresponding hypoxia directly affects platelet function. Recently, it has been shown that cardiac arrest for several minutes and following resuscitation did significantly increase platelet mitochondrial bioenergetics within 4 h in a porcine model [18] which would improve platelet activation and coagulation. On the other hand, lactate acidosis has been shown to impair platelet function in another pig model [19]. This finding might explain the negative correlation of lactate and SOFA score with collagen AUC, indicating stronger aspirin-mediated inhibition despite potentially reduced gastrointestinal absorption.

Impaired platelet inhibition might also be due to accelerated platelet turnover which has been described previously in states of inflammation [16]. The latter mechanism was also considered to be the most relevant in the small cohort of the study published by Llitjos et al. [9].

Besides impaired aspirin effects, our study also indicates a moderate effect on platelet inhibition by P2Y12 inhibitors, which is, however, only detectable for ticagrelor and prasugrel.

The question on potentially reduced inhibitory effects of crushed oral P2Y12 inhibitors is answered in three well conducted trials for each of the drugs [20–22]. These trials indicate no reduction or delay in platelet inhibition after crushing tablets but rather show an accelerated and increased mode of action.

Absorption of P2Y12 inhibitors is likely to be impaired in a setting of therapeutic hypothermia and centralized circulation. This idea is supported by a recent study directly measuring clopidogrel plasma levels in previously P2Y12-inhibitor-naïve resuscitated patients with myocardial infarction treated with therapeutic hypothermia. In this study, a significantly reduced plasma concentration for clopidogrel was detected 2 and 4 h after administration [23].

It has already been shown that cardiogenic shock itself is associated with a high prevalence of clopidogrel resistance [24]. Hypothermia itself reduces P450 clearance by 7–22% per degree Celsius [25]. Hence, reduction in body core temperature by ~ 4 °C impairs this system considerably.

Besides altered metabolism hypothermia might also influence cellular drug response affecting the potency of drugs. So far, no direct data are available with respect to P2Y12 inhibitors. However, it has been shown in vitro that impaired effect was only observed for clopidogrel but not for aspirin in an in vitro hypothermia model indicating drug and not cell specific reasons for the reduced effect [26].

In contrast to a previous report [27], the present study did not show blunted effects in clopidogrel treated post-CPR patients compared to control but reduced effect in both groups if compared to treatment with either ticagrelor or prasugrel. This is in line with a recent analysis of the IABP shock trial; there was no significant interaction regarding mortality (*p* = 0.06) between the use of mild hypothermia and the type of P2Y12 inhibitor used if prasugrel/ticagrelor was tested versus clopidogrel” [28]. In addition, it is important to note that platelet inhibition was weakest on day three although patients already reached normal body core temperature, supporting the hypothesis that not hypothermia but delayed or reduced absorption due to cardiogenic shock or temperature independent mechanisms underlie the reduced P2Y12-mediated platelet inhibition.

Except the described changes in P450 clearance, all these aspects would also influence aspirin-mediated platelet inhibition. Our data indicate that this aspect of platelet inhibition might be clinically even more important than the observed slight reduction in P2Y12-mediated inhibition which still remained within the therapeutic range in all patients, especially if the newer drugs (prasugrel, ticagrelor) were used.

Despite these data, retrospective analysis of larger cohorts failed to detect a significant increase in stent thrombosis in resuscitated patients treated with therapeutic hypothermia compared to regular ACS patients although percentages were increased in patients treated with hypothermia [29, 30].

In aspirin-mediated platelet inhibition, a different pattern was observed. On day 1, good and sufficient inhibition was detected in all patients. These values, however, are independent of gastrotintestinal absorption as all patients were initially treated intravenously by the emergency team or in the cath lab. All further applications were with crushed tablets via the gastric line, and reduced inhibitory effect could therefore either be due to lower maintenance dose [100 mg vs. initial dose 250 mg (150–300)] or rather malabsorption after gastric application. Therefore, prolonged intravenous application of aspirin might be recommended.

## Limitations of the study

Several limitations of the study have to be kept in mind interpreting the data. The number of patients in the resuscitated group is rather small, especially if effects of single drugs are evaluated. Moreover, we do not have platelet function analysis for every day in every patient since analysis was only available on working days. Lastly, the control group was heterogeneous, since these patients only got platelet function analysis once and we therefore used three different age- and gender-matched control groups for the tree first days after resuscitation. Differences between the three control groups were negligible with respect to baseline characteristics and platelet function inhibition, however, still a methodological limitation.

## Conclusion

Aspirin- and P2Y12-mediated platelet inhibition is impaired in resuscitated patients treated with therapeutic hypothermia. There are no significant differences between the three P2Y12 inhibitors in hypothermic patients, whereas the newer inhibitors are more potent under normothermic conditions but do show slightly weaker inhibition in resuscitated patients. Potentially, intravenous aspirin and intravenous P2Y12 inhibitors might still supply an even more predictable and stable platelet inhibition; however, no data in resuscitated patients are available, yet.

## Additional files

**Additional file 1.** Association of platelet function and SOFA score.

**Additional file 2.** Demographic data within the group of resuscitated patients divided by the P2Y12 inhibitor used.

## Abbreviations

ACS: acute coronary syndrome
ADP: adenosine diphosphate
AMI: acute myocardial infarction
AUC: area under the curve
CPR: cardiopulmonary resuscitation
GpIIb/IIIa: glycoprotein IIb/IIIa
IQR: inter quartile range
NSTEMI: non-ST elevation myocardial infarction
PCI: percutaneous coronary intervention
SOFA: sequential organ failure assessment score
ST: stent thrombosis
STEMI: ST elevation myocardial infarction
