# Supplementary figures and images for: Impaired aspirin-mediated platelet function inhibition in resuscitated patients with acute myocardial infarction treated with therapeutic hypothermia: a prospective, observational, non-randomized single-centre study
Source: Ann Intensive Care. 2018 Feb 21;8:28. doi: 10.1186/s13613-018-0366-x (PMC5821616; doi:10.1186/s13613-018-0366-x)

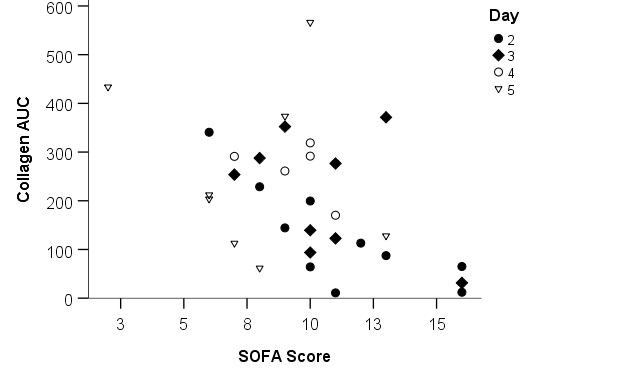


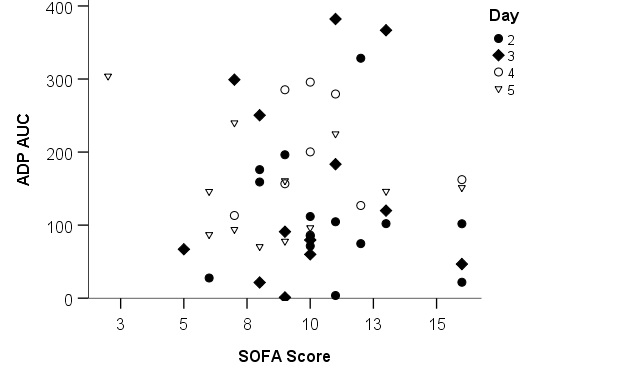

Supplement: Supplementary file 1 — Additional file 1. Association of platelet function and SOFA score. [file 13613_2018_366_MOESM1_ESM.docx]
